# Functional Implications of Marine Biosurfactants and Their Therapeutic Potential Against Alzheimer's Disease

**DOI:** 10.1002/fsn3.72106

**Published:** 2026-07-20

**Authors:** Zainab Irfan, Shakti Ketan Prusty, Sumon Giri, Arijit Nandi, Mostafa Gouda

**Affiliations:** ^1^ Department of Pharmacology, School of Pharmaceutical Sciences Siksha ‘O’ Anusandhan (Deemed to Be University) Bhubaneswar Odisha India; ^2^ Department of Pharmaceutical Technology Brainware University Kolkata West Bengal India; ^3^ Department of Pharmacy Sanaka Educational Trust Group of Institutions (SETGOI) Durgapur West Bengal India; ^4^ College of Biosystems Engineering and Food Science Zhejiang University Hangzhou China; ^5^ Department of Nutrition & Food Science National Research Centre Giza Egypt

**Keywords:** Alzheimers disease, amphiphilic, antioxidant, biosurfactant, neuroprotective

## Abstract

Neuronal damage and cerebral impairment are defining characteristics of Alzheimer's disease (AD), a progressive neurodegenerative disorder associated with amyloid‐β aggregation, oxidative stress, and neuroinflammation. Current treatments mainly provide symptomatic relief and do not effectively prevent disease progression, highlighting the need for novel disease‐modifying strategies. Marine microorganisms produce amphiphilic substances called biosurfactants that exhibit diverse biological properties, including anti‐inflammatory, antioxidant, and neuroprotective activities. Due to their amphiphilic nature, biosurfactants may interact with cellular membranes and modulate crucial pathological processes in AD, including inhibiting amyloid‐β fibrillation, reducing oxidative stress, suppressing neuroinflammation via pathways such as NF‐κB signaling, and enhancing drug delivery across the blood–brain barrier. Notably, various biosurfactant classes, such as lipopeptides, exhibit significant neuroprotective potential. This review explores the structural diversity and biological functions of marine biosurfactants, their potential as therapeutic candidates for managing AD, and the pathophysiology of AD. Overall, marine biosurfactants represent promising and sustainable therapeutic options for AD; however, extensive experimental and clinical research is necessary to confirm their effectiveness.

## Graphical abstract

Marine‐derived biosurfactants produced by microorganisms (including bacteria, fungi, and yeast) have amphiphilic properties that facilitate membrane interactions and transport across the blood–brain barrier (BBB). These biosurfactants exhibit neuroprotective activity by targeting mechanisms implicated in Alzheimer's disease, including reducing oxidative stress, inhibiting amyloid‐β aggregation, suppressing tau hyperphosphorylation, attenuating neuroinflammation, preventing ferroptosis, and protecting mitochondria. These actions support neuronal survival, maintain synaptic integrity, improve cognition, and facilitate drug delivery to the brain, making them promising natural candidates for Alzheimer's treatment.

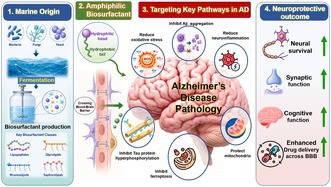

## 1 Introduction

Alzheimer's disease is a complex and rapidly progressing neurodegenerative disease caused by multiple pathological processes. These include amyloid‐β protein (Aβ) accumulation in fibrillar plaques, tau protein aggregation in neurofibrillary tangles, neuroinflammation, neurodegeneration, oxidative cellular injury, mitochondrial dysfunction, autophagy abnormalities, bioenergetic and metabolic changes, synaptic dysfunction, vascular disturbances, and epigenetic changes (DeTure and Dickson 2019; Jack et al. 2018; Scheltens et al. 2021). Older adults are most often impacted by AD, which is commonly recognized as the most prevalent cause of dementia (Uddin et al. 2020). About 24.3 million individuals worldwide are struggling with AD, rendering it a major global health issue. The prevalence of dementia, including AD, is expected to almost double every two decades as the population ages. An estimated 78 million dementia cases are predicted by 2030, and by 2050, this estimate is forecast to rise to around 139 million (Leverton and Pui Kin Kor 2023). This growing global burden emphasizes the urgent need for effective preventive and therapeutic measures.

There are currently no pharmacotherapeutic drugs that are successful in treating or preventing AD. The use of medications that decrease glutamate levels and increase acetylcholine levels (cholinesterase inhibitors) is the only effective way to normalize neurotransmitter levels in AD (Colovic et al. 2013; Conway 2020). Each of these compounds has varying potency depending on the patient and the stage of the illness, and these medications may cause side effects such as nausea, vomiting, and diarrhea. For these reasons, several efforts have been made to create novel therapeutic agents to identify effective medications to treat AD (Alvarino et al. 2019; Barbalace et al. 2019; Rahman et al. 2021).

An abundance of structurally varied bioactive compounds with significant pharmacological and nutraceutical potential can be found in marine habitats. To survive harsh environments, numerous marine species produce specialized secondary metabolites that have garnered increasing attention for their biological activities. Because of their many biological activities and functional characteristics, biosurfactants—amphiphilic compounds made by microorganisms—have drawn the most interest among these metabolites (Barzkar et al. 2019; Ciccone et al. 2021; Martins et al. 2020).

In addition to few animals and plants, an array of microorganisms, including bacteria (*Acinetobacter* sp., *Bacillus* sp., and *Pseudomonas* sp.), filamentous fungi (*Aspergillus* sp., *Fusarium* sp., *Penicillium* sp., *Trichoderma* sp., *Ustilago* sp.) and yeast (*Kluyveromyces*, *Pseudozyma*, *Rhodotorula*, *Torulopsis*, *Saccharomyces*, *Candida*), produce biosurfactants (BS) (Fenibo et al. 2019; Sena et al. 2018). These compounds can reduce surface tension and form emulsions because they have both hydrophilic and hydrophobic moieties (Ceresa, Rinaldi, et al. 2021; Sharma et al. 2021). Due to their amphiphilic structure, biosurfactants exhibit a wide range of physicochemical properties, including high surface activity, a low critical micelle concentration, and the ability to form microemulsions, humectants, foams, and cleaning agents (Sarubbo et al. 2022). Significantly, biosurfactants have attracted interest in food science and biotechnology, as well as in pharmaceutical research, since they can function in food systems as natural emulsifiers and stabilizing agents. In addition to their use in food processing, biosurfactants possess biological activities including antioxidant and anti‐inflammatory effects (Irfan et al. 2024; Naughton et al. 2019). These bioactivities imply that biosurfactants might also be used in functional foods or nutraceutical formulations that promote health. Their anti‐inflammatory and antioxidant qualities are highly relevant to neurodegenerative diseases like Alzheimer's, where oxidative stress and neuroinflammation are important factors in the development of the disease. Given these properties, marine‐derived biosurfactants have emerged as potential candidates for developing novel therapeutic or nutraceutical approaches to mitigate AD pathology. Major scientific databases such as PubMed, Scopus, Web of Science, ScienceDirect, and Google Scholar were used to do a thorough literature search. A combination of keywords, including “marine biosurfactants,” “microbial biosurfactants,” “neuroprotective activity,” “Alzheimer's disease,” “oxidative stress,” and “anti‐inflammatory activity,” was used in the search strategy. Peer‐reviewed publications in only the English language were considered. To maintain scientific rigor and relevance, articles unrelated to biosurfactants, non‐degenerative uses, conference abstracts, or non‐peer‐reviewed sources were excluded.

This review provides a comprehensive discussion of the potential of biosurfactants as a novel strategy for AD treatment. It provides a thorough overview of biosurfactants, including their structural diversity and biological activities, followed by a discussion on the pathogenesis of AD.

## 2 Biosurfactants: Sources, Properties and Therapeutic Applications

Microbially derived bioactive compounds with surface‐active properties are known as biosurfactants and can be used successfully in place of synthetic surfactants (Thakur et al. 2024). These substances have a hydrophilic tail and a hydrophobic head, rendering them amphiphilic. The hydrophilic part of the substances typically includes cyclic peptides, phosphates, carboxylic acids, or alcoholic groups, while the hydrophobic zone comprises long‐chain fatty acids, hydroxyl fatty acids, α‐alkyl‐β‐hydroxyl fatty acids, and more (Santos et al. 2016). Biosurfactants are made from renewable substrates such as molasses, glycerol, whey, and plant oils, enabling eco‐friendly, cost‐effective production, exhibiting substantial surface activity, high specificity, and the capacity to work in harsh environments (Ibrahim 2018). The application of microbial surfactants is encouraged in the majority of industries owing to greater efficacy and benefits over chemical ones, particularly in the areas of biodegradability, favorable production conditions, ecological affinity, reduced toxicity, greater selectivity, and precise action over extreme temperatures, pH, and salinities (Hrůzová et al. 2020; Rincón‐Fontán et al. 2020).

Based on their molecular structure and functional roles, biosurfactants are classified into high‐molecular‐weight and low‐molecular‐weight groups. Low‐molecular‐weight biosurfactants, including glycolipids such as fructose lipids, sophorolipids, and trehalose, and peptidyl lipids such as surfactin, polymyxin, and other substances, can effectively reduce surface and interfacial tension (Goussous et al. 2017; Roy et al. 2016; Wu, Liu, et al. 2019). According to reports, surfactin, a biosurfactant made by *Bacillus subtilis* ( *B. subtilis* ), may be employed therapeutically to treat AD and other neurodegenerative conditions involving neuroinflammation (Park et al. 2013a). On the other hand, High‐molecular‐weight biosurfactants, also known as bioemulsifiers, are composed of polymeric and particulate substances, such as emulsan, alasan, and liposan, and are mainly involved in the stabilization of emulsions rather than in surface tension reduction (Tian et al. 2019). *Arthrobacter* sp., *B. subtilis* , *Pseudomonas aeruginosa* ( *P. aeruginosa* ), *Acinetobacter calcoaceticus* ( *A. calcoaceticus* ), *Candida lipolytica* ( *C. lipolytica* ), *Corynebacterium* sp., *Nocardia* sp., *Bacillus licheniformis* ( *B. licheniformis* ), and several other species are the origin of most of the biosurfactants (Akbari et al. 2018; Martins et al. 2018).

### 2.1 Biosurfactant‐Producing Marine Microorganisms

Microorganisms that produce biosurfactants can be found in various types of water, notably freshwater, groundwater, and seawater, as well as in environments with extreme temperatures, salinity, and pH (such as oil reservoirs and hypersaline sites) (Nikolova and Gutierrez 2021). These unique environmental factors offer marine environments a valuable opportunity to discover novel microorganisms that produce biosurfactants. However, it must be noted that most of the marine microbial diversity remains unknown, potentially due to the inability to cultivate unculturable marine microorganisms in laboratory environments (Gudiña et al. 2016). The majority of microbial biosurfactants' chemical structure versatility allows a broad range of uses, particularly in the healthcare sector (Bae et al. 2019), food (Maniglia et al. 2019), agriculture (Shah and Daverey 2021), pharmaceutical (Chuo et al. 2019), biomedicine (Guerfali et al. 2019), materials engineering (Ranjana et al. 2019), bioenergy (Menon et al. 2010), and bioremediation (Ye et al. 2016). Table 1 summarizes the marine microorganisms that produce biosurfactants.

**TABLE 1:** Biosurfactant‐producing microorganisms from marine environments.

Microorganisms	Microbial species	Types of biosurfactant	References
Bacteria	*Alcanivorax dieselolei*	Glycolipid	(Hassanshahian et al. 2012)
Bacteria	*Paracoccus* sp. MJ9	Rhamnolipid	(Xu et al. 2020)
Bacteria	*Corynebacterium kutscheri*	Glycolipopeptide	(Thavasi et al. 2011)
Bacteria	*Idiomarina* sp.185	Glycolipid	(Malavenda et al. 2015)
Bacteria	*Bacillus* sp. CS30	Surfactin	(Wu, Lai, et al. 2019)
Bacteria	*Bacillus* sp. BS3	Lipopeptide	(Donio et al. 2013)
Fungi	*Cyberlindnera saturnus* SBPN‐27	Cybersan	(Balan et al. 2016)
Fungi	*Aspergillus terreus* MUT 271 and *Trichoderma harzianum* MUT 290	Cerato‐platanins	(Pitocchi et al. 2020)
Fungi	*Ustilago maydis*	Cellobiose lipids	(Valkenburg et al. 2025)
Fungi	*Ustilago maydis* FBD12	Glycolipids	(Villagrán et al. 2023)
Yeast	*Starmerella bombicola*	Sophorolipids	(Lodens et al. 2020)
Yeast	*Saccharomyces cerevisiae* URM 6670	Glycolipid	(Ribeiro et al. 2020)

### 2.2 Classification of Biosurfactants

**FIGURE 1:**
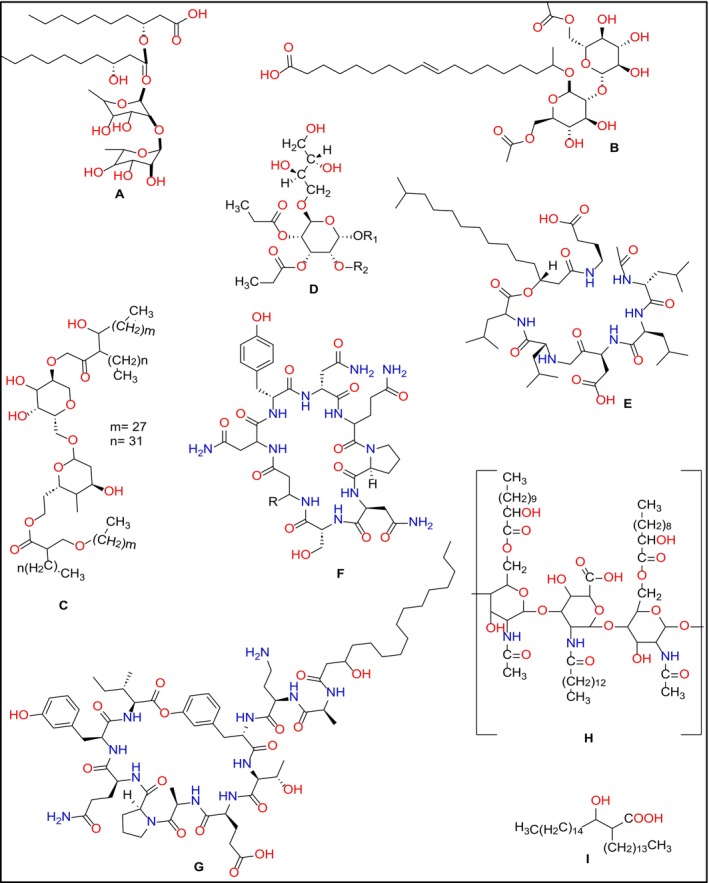
Biosurfactants produced from marine microorganisms (A) Rhamnolipids; (B) Sophorolipid; (C) Mannosylerythrol lipids; (D) Trehalolipid; (E) Iturin; (F) Surfactin; (G) Fengycin; (H) Emulsan; (I) Corynomycolic acid.

Biosurfactants with amine groups are cationic, whereas most are neutral or anionic. Long‐chain fatty acids make up the hydrophobic moiety, while alcohol, phosphate, carboxyl acid, carbohydrate, cyclic peptide, or amino acid might make up the hydrophilic moiety. The molar mass of biosurfactants usually ranges from 500 to 1500 Da (Sharma et al. 2023). Although the chemical composition of biosurfactants varies substantially among microbial species, they can be generically classified by their molecular weight or chemical charge (Eras‐Muñoz et al. 2022; Vijayakumar and Saravanan 2015). Table 2 provides examples of the major classes of microbial biosurfactants, such as polymeric biosurfactants, fatty acids, neutral lipids, phospholipids, glycolipids, lipopeptides, and lipoproteins (Figure 1).

**TABLE 2:** Major classes of microbial biosurfactants.

Biosurfactant molecular class	Biosurfactant	Chemical structure	References
Low‐molecular‐weight biosurfactants			
Glycolipids	Rhamnolipid	A β‐glycosidic bond connects the rhamnose monosaccharide or monosaccharides to the 3‐hydroxyl fatty acid unit	(Salazar‐Bryam et al. 2021)
	Sophorolipid	Both lactonic and acidic forms of the dimeric sugar sophorose are linked to the head by a long‐chain hydroxy fatty acid tail	(Liu et al. 2019)
	Trehalose lipids	Trehalose sugar is connected to C_20_ to C_90_ long‐chain fatty acids	(Pinto et al. 2018)
	Mannosylerythrol lipids (MELs)	Long‐chain fatty acids are connected to a hydrophilic head group, a mannopyranosyl‐meso‐erythritol	(Niu et al. 2019)
Lipopeptides and lipoproteins	Surfactin	Cyclic lipopeptide is composed of C_13_–C_16_ hydroxy fatty acid chain and heptapeptides	(Yang et al. 2020)
	Iturin	A cyclic lipopeptide is formed when seven peptides are bound together by a chain of β‐hydroxy fatty acids	(Yaraguppi et al. 2023)
	Fengycin	Consists of an inner ester bond connecting cyclic decapeptides to a chain of β‐hydroxy fatty acids	(Qi et al. 2025)
High‐molecular‐weight bioemulsifiers			
Polymeric biosurfactant	Emulsan	A protein with a lipoheteropolysaccharide (apoemulsan) combination	(D'Almeida et al. 2024)
	Alasan	A combination of high molecular mass proteins and anionic polysaccharides rich in alanine	(Annuar et al. 2023)
	Liposan	Heteropolysaccharides and protein complex	(Srivastava et al. 2022)

### 2.3 Marine Glycolipids

Among biosurfactants, glycolipids are the most commonly used kind. They consist of one or more fatty acids, hydroxy fatty acids, or fatty alcohols linked to one or more carbohydrates. A variety of glycolipid biosurfactants, including mannosylerythritol lipids (MELs), rhamnolipids, trehalolipids, and sophorolipids, consist of mono‐ and di‐saccharides in conjunction with hydroxy‐ or long‐chain aliphatic acids (Drakontis and Amin 2020). Among the most well‐known microorganisms responsible for the production of glycolipids are *Pseudomonas* species, *Burkholderia* species, *Rhodococcus* species, *Candida* species, *Arthrobacter* species, *Starmerella bombicola, Corynebacterium* species, *Mycobacterium* species, and *Candida antartica* (Aslam et al. 2023; Rawat et al. 2020). With their ability to emulsify hydrocarbons, these glycolipid biosurfactants are used in medicine for their antibacterial, anti‐inflammatory, and immune‐system‐regulating activities, while in cosmetics they are employed for their wetting and moisturizing properties. They are also used for oil/water emulsion stabilization (Gürkök and Özdal 2021; Pal et al. 2023).

### 2.4 Marine Lipopeptides

Lipopeptide biosurfactants are cyclic entities with an array of molecular functions and structures that predominantly originate from *Bacillus* sp. and *Pseudomonas* sp. species. Lipopeptides consist principally of hydrophilic peptides linked to hydrophobic lipids and fatty acids. The families of surfactin, iturin, and fengycin constitute the majority of the lipopeptides that *Bacillus* produces. They can be identified based on their fatty acid types, peptide length and cyclization, and amino acid sequence (Bartal et al. 2018; Dini et al. 2024). Surfactin, a common lipopeptide from *B. subtilis* , has been extensively studied owing to its distinctive emulsification, antimicrobial properties, and surface activity (Nelson et al. 2020; Santos et al. 2018). In comparison to *Bacillus*‐derived lipopeptides such as surfactin, lipopeptides produced from *Pseudomonas*, such as viscosin and putisolvin, have high surface and biological activity and are composed of a short oligopeptide linked to fatty acid tails (Mnif and Ghribi 2015). It has been reported that these lipopeptide biosurfactants, such as surfactin, iturin, viscosin, and putisolvin, possess thrombolytic, antiviral, antibacterial, anti‐inflammatory, and antimycoplasma properties (Götze and Stallforth 2020).

### 2.5 Marine Fatty Acids and Phospholipids

The phosphatidylethanolamine‐rich material produced by a range of bacteria and yeasts, including Acinetobacter sp. and *Thiobacillus trioxidanes*, dissolves n‐alkanes in water, forming optically transparent microemulsions. Fatty acids and phospholipids, which are considered biosurfactants, are by‐products of microbial oxidation of alkanes. Fatty acids are widely employed in the food industry, but because of their membrane section, phospholipids have been used in gene carrier systems. Two examples of phospholipid biosurfactants are lecithin and lysolecithin (Al‐Hawash et al. 2018; McClements and Gumus 2016; Sarubbo et al. 2022).

### 2.6 Marine Polymeric Biosurfactants

Emulsan, liposan, alasan, and mannoprotein, the well‐known polymeric biosurfactants, have strong bioemulsifying properties (Vandana and Singh 2018). For example, bacteria, yeast, and fungi create bioemulsifiers (BE), which are high‐molecular‐weight compounds. They are formed of amalgams of proteins, lipoproteins, heteropolysaccharides, and lipopolysaccharides that can either be released or adhere to the cell surface (Uzoigwe et al. 2015). One of the earlier recognized species to produce bioemulsifiers is *Acinetobacter* spp. The two most well‐known commercial bioemulsifiers produced by *Acinetobacter* spp. are emulsan and alasan (Mujumdar et al. 2019).

### 2.7 Marine Physicochemical Properties

**FIGURE 2:**
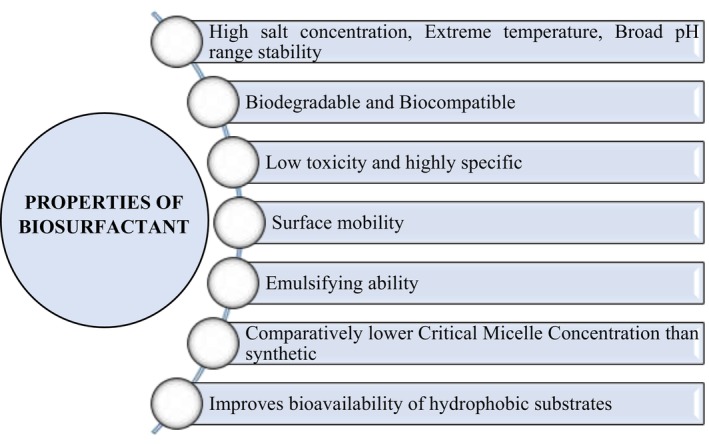
Properties of biosurfactant.

The enhanced attributes of biosurfactants over chemically synthesized alternatives and their adaptability to a broad spectrum of substrates render them economically viable. Microbial surfactants have been defined by their surface mobility, stability against changes in pH, temperature, and ionic quality, biodegradability, low toxicity, emulsifying and demulsifying properties, and antibacterial properties (Figure 2) (Kumar et al. 2021). Some of the key characteristics of biosurfactants are discussed in the next section.

## 3 Marine Biosurfactants' Physicochemical Properties

### 3.1 Surface and Interfacial Tension

It is possible to reduce surface tension and interfacial pressure using surfactants. Many biosurfactants have been reported to be more effective than chemical surfactants, and their Critical Micelle Concentration (CMC) is significantly lower, indicating that less surfactant is required to reduce surface strain (Markande et al. 2021). At a concentration of 20 μM, surfactin effectively reduces water surface tension from 72 mN/m to 27 mN/m. In terms of their aggregation activity, the CMC of surfactin decreases with longer fatty acid chains (Zhen et al. 2023).

### 3.2 Emulsifying Agents

Synthetic surfactants can accumulate and cause ecological and health issues because they do not readily break down in the environment. Their inability may disrupt normal microbial populations and pose a hazard to aquatic life. Biosurfactants, on the other hand, are safer than traditional chemical surfactants because they are biodegradable and eco‐friendly. Biosurfactants are being investigated for use in the food, pharmaceutical, and biochemical industries because of their natural origin, low toxicity, and high biodegradability (Bhatt et al. 2022; Zhang et al. 2022).

### 3.3 Low Toxicity

Biosurfactants usually exhibit much lower toxicity than traditional synthetic surfactants due to their natural origin, biodegradability, and better biocompatibility. They are desirable alternatives for pharmaceutical, cosmetic, culinary, and drug‐delivery applications because of their amphiphilic structure, which facilitates selective interaction with biological membranes while reducing irreversible membrane disruptions, bioaccumulation, and unfavorable environmental impacts. Several in vitro and in vivo studies have reported that glycolipids and lipopeptides exhibit comparatively low cytotoxicity at concentrations useful for biological activity. For instance, rhamnolipids and sophorolipids have shown low toxicity to mammalian cell lines while retaining their antibacterial and anti‐inflammatory properties. Biosurfactants have a better safety profile than synthetic surfactants like commercial Corexit (composed of anionic surfactant DOSS and non‐ionic surfactants Span 80, Tween 80, and Tween 85), according to comparative toxicity studies. While retaining similar or improved surface‐active properties, these biosurfactants exhibit decreased cytotoxicity, increased biodegradability, decreased ecotoxicity, and reduced oxidative stress and inflammatory responses. Collectively, these attributes support the emergence of biosurfactants as safer and more environmentally friendly substitutes for synthetic surfactants in pharmaceutical, biomedical and drug delivery applications (Gofstein and Leigh 2023; Mallik and Banerjee 2022; Sharma et al. 2022; Sen et al. 2019; Zouari et al. 2016).

### 3.4 Thermophilic

Biosurfactants are more desirable than chemical surfactants owing to their capacity to withstand changes in pH and temperature. It is widely recognized that biosurfactants can withstand a broad range of pH and temperature variations. Lichenysin, which is made from *B. licheniformus*, is resistant to temperatures up to 50°C and to a pH range between 4.5 and 9 (Sałek et al. 2022). According to another study, the biosurfactant made by *A. protophormiae* was robust to both pH and temperature, with tolerances of 2–12 and 30°C–100°C, respectively (Barbosa et al. 2022).

### 3.5 Solubilization

Self‐assembling amphiphiles in aqueous solutions can dissolve hydrophobic substances (like oil) that preferentially reside in the hydrophobic realm of the amphiphilic nanostructure. When biosurfactants are present, concentration, pH, and the addition of salts and electrolytes, which change micelle size, all affect the solubility of hydrophobic organic molecules (Jahan et al. 2020).

Rhamnolipids can enhance the solubility of hydrophobic compounds by making amphiphilic molecules more hydrophobic. With rising pH, biosurfactant molecules tend to form micelles and vesicles, which restrict the solubilization of other molecules. The substrate‐specific characteristics of biosurfactants include the ability to solubilize or emulsify different hydrocarbons at different rates (Shao et al. 2017). Compared to synthetic surfactants such as sodium dodecyl sulfate, biosurfactants are more efficient solubilizing agents (López‐Prieto et al. 2020).

### 3.6 Pharmaceutical Applications

**FIGURE 3:**
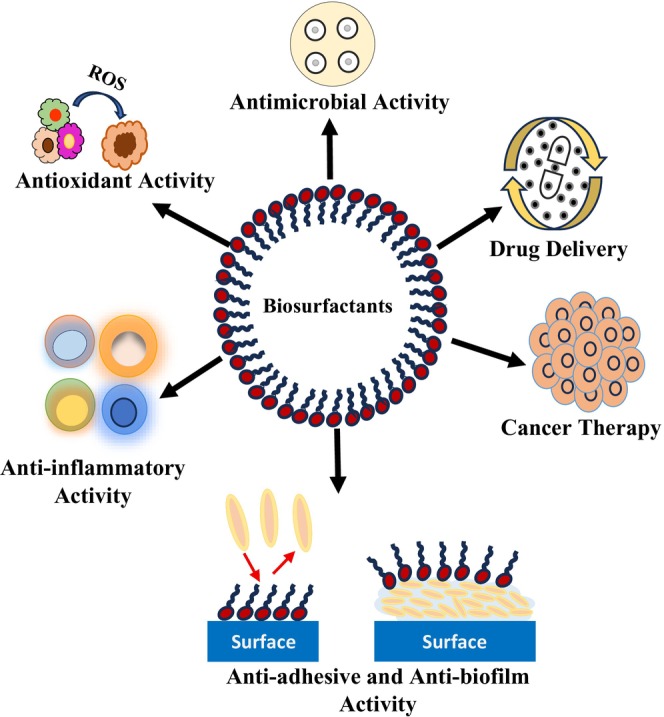
Application of biosurfactants in the pharmaceutical industry.

Numerous biological attributes, including antibacterial, anti‐adhesive, anti‐biofilm, anti‐cancer, antiviral, drug‐delivery, and others, have been reported for several biosurfactants as presented in Figure 3.

### 3.7 Anti‐Adhesive and Anti‐Biofilm Activities

Biosurfactants are deemed more suitable anti‐adhesive agents by changing their hydrophobicity, which aids in reducing the adherence of microorganisms to a biofilm's surface. Because the surface of the biofilm is not influenced by external factors such as microorganisms, hydrophobicity, or electrical charges, biosurfactants' characteristics aid in the formation of a stable biofilm (Haddaji et al. 2022). Due to their high correlation with antibiotic resistance and chronic healthcare‐associated infections (HAIs), biofilms are a major issue in biomedical research (Haque et al. 2018; Sharma et al. 2019). By reducing microbial cell viability and microbial adhesion, biosurfactants suppress biofilm formation (Banat et al. 2021; Ceresa, Rinaldi, et al. 2021). In addition to their antimicrobial properties, biosurfactants' capacity to form voids in the biofilm structure and disrupt quorum‐sensing signaling and gene expression is also linked to their anti‐biofilm activity (Haque et al. 2016; Ibacache‐Quiroga et al. 2013). Cordeiro et al. (2018) observed that TIM96, a combination of surfactin, iturin, and fengicin, reduced the thickness and cell viability of mature biofilms by up to 99.2% and prevented cell adhesion by up to 96.89% when co‐incubated with *Trichosporon* sp.

## 4 Marine Biosurfactants' Functional Bioactivity

### 4.1 Antimicrobial Activity

A multitude of microorganisms that have evolved to survive in extreme environments can be found in the vast and diverse marine ecosystem. These microorganisms may also produce a range of unique bioactive substances, including novel biosurfactants with strong antimicrobial properties that can effectively combat microbial infections. Utilizing biosurfactants produced by marine microorganisms not only helps conserve and explore marine biodiversity but also provides opportunities to develop sustainable, alternative antimicrobial agents (Kubicki et al. 2019). Microbial metabolites are a primary source of bioactive compounds in these circumstances. Several biosurfactants, including daptomycin and the echinocandins caspofungin, micafungin, and anidulafungin, possess potent antibacterial and antifungal properties and have previously achieved commercial antibiotic status, making them particularly appealing (Ceresa, Fracchia, et al. 2021).

For biosurfactants with antimicrobial properties, lipopeptides and glycolipids are the most commonly proclaimed types (Cochrane and Vederas 2016). Specifically, Polymyxin A and Polymyxin B from *Bacillus polymyxa* (Ardebili et al. 2023); surfactin, iturin, fengycin, mycosubtilins, and bacillomycins produced by *Bacillus subtilis* (Ali et al. 2022); pumilacidin produced by *Bacillus pumilus* (Dasgupta et al. 2023); lichenysin from *Bacillus licheniformis* (Zammuto et al. 2023); and viscosin from *Pseudomonas fluorescens* (Chauhan et al. 2023) are well known as antimicrobial lipopeptides. The most explored glycolipids include rhamnolipids from *Pseudomonas aeruginosa* (Cerqueira dos Santos et al. 2024), sophorolipids from *Candida bombicola* (Cho et al. 2022), and mannosylerythritol lipids from *Candida antarctica* (Liu et al. 2024).

According to Mu et al. (2018), the biosurfactant‐producing lactobacilli may play a probiotic role in the preservation and repair of healthy intestinal and urogenital tracts, as well as in the defense of the skin and gastrointestinal (GI) systems (De Giani et al. 2021). They also offered protection against infections and proposed a dependable alternative to antibiotics for treatment and prevention. In the study by Manikkasundaram et al. (2023), the glycolipid HRB1 was examined for its antifungal activity against *Magnaporthe grisea* and *Alternaria* spp., its anti‐biofilm activity against *P. aeruginosa*, and its potential to block *C. violaceum* quorum‐sensing signaling. The glycolipid's anti‐phytofungal, anti‐biofilm, anti‐quorum‐sensing, antioxidant, anticancer, and dye‐degradation properties were determined by the researchers.

### 4.2 Anticancer Activity

Cancer continues to be the most common cause of death globally, despite tremendous advancements in cancer treatment. In 2020, the World Health Organization (WHO) released data that showed an expected 10 million deaths and 19.3 million new cases. While a recent analysis projects that for the year 2022, there were close to 20 million new cases of cancer, with 9.7 million deaths from cancer (Bray et al. 2025; Sung et al. 2021). Over the years, novel cancer therapies have continuously blossomed to counter this deadly disease; nonetheless, the primary cancer therapies available remain radiotherapy and chemotherapy (Pucci et al. 2019). The lack of specificity of anticancer medications for cancer cells is the reason for the increase in mortality rates, notwithstanding a few benefits of these therapies. This leads to painful side effects, low success rates, and cancer cells developing resistance to multiple therapies (Mansoori et al. 2017). Investigations into natural anticancer medications as potential alternatives to chemical medications are necessary to address the shortcomings of chemical medications. Bacteria, in particular, have garnered considerable attention as potential sources of new anticancer chemicals due to their low toxicity, high selectivity, and biodegradability (Dan et al. 2021). Additionally, biosurfactants have recently demonstrated potential as alternative agents to treat several cancer types, including cervical, oral, colon, lung, liver, breast, and pancreatic cancers (Pilz et al. 2023). Since they can control specific processes in mammalian cells, they have shown promise in cancer treatment by inhibiting aberrant disease progression through suppression of cell migration, viability, and proliferation (Bjerk et al. 2021). The impact of glycolipids, namely acidic and lactonic sophorolipids, bolalipids, and glucolipids, on cancer cells was examined by Haque et al. (2021). The studies employed three well‐known cell lines: the mouse skin melanoma cell line (B16F10), the lung cancer cell line (A549), and the breast cancer cell line (MDA‐MB‐231). The results indicate that both lactonic sophorolipids and glucolipids inhibit cancer cell motility, possibly by interfering with actin filaments and inducing reactive oxygen species production. Furthermore, these biosurfactants altered the mitochondrial membrane potential, ultimately leading to necrosis‐induced cell death (Haque et al. 2021).

### 4.3 Antioxidant Activity

The antioxidant properties of biosurfactants have attracted interest from various industries in recent years, as natural compounds are more prevalent and offer greater advantages than synthetic antioxidants. Reactive oxygen species (ROS) and reactive nitrogen species (RNS) are effectively suppressed by antioxidants, which help prevent cancer, heart disease, and neurodegenerative diseases (Nadaf et al. 2021). Mannosyl erythritol lipids (MELs) are multipurpose biosurfactants that possess biochemical and interfacial properties. MEL‐C exhibits the strongest antioxidant activity and can scavenge free radicals. As a result, it offers the best protection and is widely utilized in the production of skincare cosmetics (Coelho et al. 2020).

### 4.4 Anti‐Inflammatory Activity

Phospholipase A_2_ (PLA_2_) participates in the secretion of arachidonic acid (AA). The different forms of PLA_2_ are collectively referred to as cytosolic phospholipase A2 (cPLA2). The release of AA, which is converted into inflammatory mediators, triggers an inflammatory response and serves as a precursor to eicosanoid secretion, which sustains the inflammatory process. Toll‐like receptor 2 (TLR‐2) can detect the structural features of biosurfactants that interact with cell membranes and macromolecules to suppress cPLA_2_ and instigate anti‐inflammatory responses (Subramaniam et al. 2020). Sophorolipids from *Candida bombicola* reduced the mRNA expression of TLR‐2, interleukin‐6 (IL‐6), and signal transducer and activator of transcription‐3 (STAT3), immunoglobulin E (IgE) levels, and lung inflammation (Das et al. 2022; Daverey et al. 2021). Accordingly, the study disclosed that sophorolipids function as anti‐inflammatory agents and as potential therapeutic compounds by downregulating IgE‐coding genes (Sajid et al. 2020). These investigations indicate that biosurfactants from microbial species exhibit anti‐inflammatory activity and are proposed as potential therapeutic candidates for the treatment of inflammatory diseases.

### 4.5 Drug Delivery Agents

The distinct surface‐active properties and advantages of biosurfactants make them ideal for use in drug delivery. Overall, considering their physicochemical properties, using biosurfactants in drug delivery shows promise for improving drug solubility, achieving controlled release, guaranteeing biocompatibility, providing protection, and enabling targeted distribution. Because of these advantages, biosurfactants are desirable substances for creating innovative and efficient medication delivery methods (Ceresa et al. 2023). A study by Lassenberger et al. (2021) developed silk‐based composite hydrogels incorporating anionic biosurfactant assemblies (sophorolipids, SL‐C18:0 and SL‐C18:1) to enhance the properties of silk fibroin. Adding sophorolipid assemblies accelerated silk fibroin (SF) gelation, suggesting a viable means to improve the mechanical and functional characteristics of SF‐based hydrogels. These advancements present encouraging opportunities for tissue engineering, controlled cell culture applications, and medication administration (Lassenberger et al. 2021). The development and synthesis of eco‐sustainable bioactive nanoparticles have demonstrated that biosurfactants are a very promising alternative to synthetic surfactants. Because of their antibacterial properties, drug transport, controlled release, and anticancer activity, they show strong potential for biomedical applications (Sharma et al. 2021, 2023). Liposomes are lipid‐based nanoparticles that are used in medicine for a variety of purposes, including medication delivery. Biosurfactants have been increasingly used in place of PEG‐lipids, which may cause hypersensitivity reactions (Chen and Zhang 2023). Liposomes can be produced by using glycolipids, particularly rhamnolipids, as demonstrated by rhamnolipid‐modified curcumin‐loaded liposomes (Cheng et al. 2019). Several benefits can be obtained by adding biosurfactants to liposome compositions. Biosurfactants can enhance liposome stability and integrity by interacting with the phospholipid bilayer, reducing interfacial tension, and preventing aggregation. Glycolipid biosurfactants such as rhamnolipids can improve controlled release of therapeutic drugs, encapsulation effectiveness, and membrane integrity. Additionally, the toxicity and hypersensitivity reaction associated with synthetic surfactants may be reduced by their biocompatibility and biodegradability properties (Müller et al. 2017).

## 5 Marine Biosurfactants: Pathophysiological Implications on Alzheimer's Disease

**FIGURE 4:**
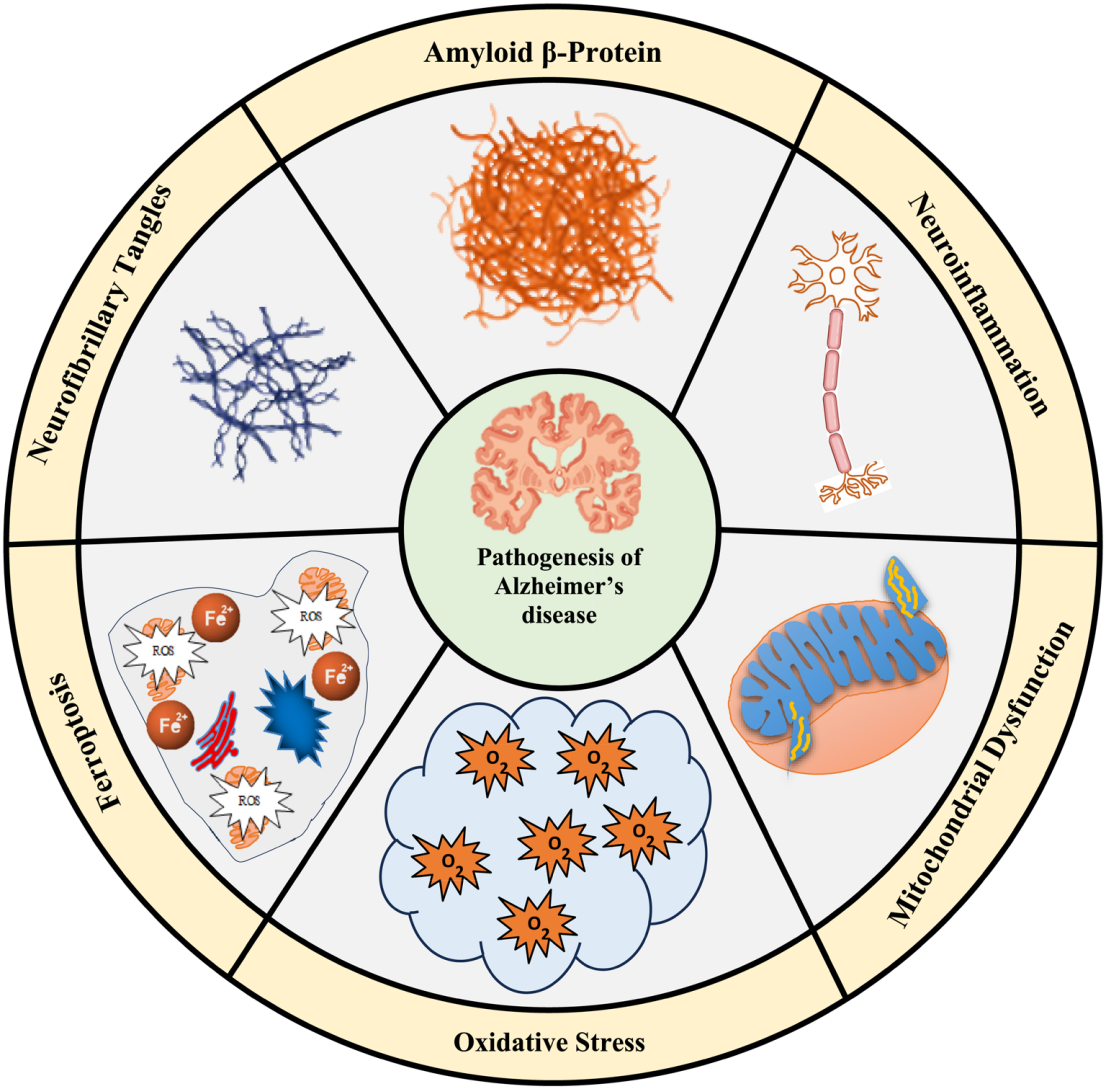
Different factors contributing to the pathogenesis of Alzheimer's disease.

Alzheimer's disease is the most widespread neurodegenerative illness with a complex, varied, and irreversible etiology that occurs with aging. This neurological condition is associated with a persistent decrease in memory and cognitive abilities. AD is becoming more common worldwide, which poses major challenges for societies and the healthcare system (Devi 2023). The actual etiology and pathogenesis of AD remain unknown, despite growing understanding of the disease's molecular, biochemical, and cellular mechanisms. This makes it more difficult to develop effective new drugs that can alter the condition (Du et al. 2018). The key pathogenic characteristics remain the formation of intracellular neurofibrillary tangles composed of aberrantly phosphorylated tau, excessive deposition of extracellular beta‐amyloid (Aβ) plaques, and neuronal death. Despite the genetic predisposition, there is mounting evidence that the fundamental causes of AD include inflammation, gliosis, changes in the ratio of ROS production to removal, mitochondrial dysfunction, and excessive accumulation of metal ions (Figure 4) (Guo et al. 2020; Ibrahim and Gabr 2019). The following sections outline the most crucial pathological processes associated with the disease.

### 5.1 β‐Amyloid Cascade Hypothesis

The main factor responsible for the pathophysiology of AD is Aβ, the primary proteolytic fragment generated by cleavage of amyloid precursor protein (APP), according to the amyloid cascade theory. Somatodendritic and axonal compartments of neurons contain APP, a single‐pass type I transmembrane protein that is characterized by a short cytoplasmic tail and a large extracellular domain (Knopman et al. 2021; Savelieff et al. 2019; Simunkova et al. 2019). The extracellular domain of APP facilitates connections between neurons by modulating cell‐to‐cell adhesion. It is anticipated that APP homodimers serve as cell‐surface G‐protein coupled receptors that bind Aβ and stimulate calcium channels to regulate neuronal signaling and neurotransmitter release (Ma et al. 2022; Richter et al. 2018).

The three proteolytic secretase enzymes, α‐, β‐, and γ‐secretase, are primarily responsible for APP processing. The potential α‐secretases include ADAM9, 10, and 17. The γ‐secretase comprises four essential elements: presenilins (PS1 and PS2), nicastrin, PEN2, and APH1. BACE1 is the principal β‐secretase in the brain (Kepp et al. 2023).

A tiny p3 fragment having no detrimental effect is released into the extracellular space when C83 is further cleaved by γ‐secretase, leaving the remaining APP intracellular domain in the cytoplasm (Guo et al. 2020; Monteiro et al. 2023). β‐secretase and the γ‐secretase complex sequentially cleave APP as part of the amyloidogenic process. The sAPPβ ectodomain is liberated after β‐cleavage, and γ‐secretase can cleave the 99 amino acid APP carboxy‐terminal fragment (also known as β‐CTF or C99) at different locations. When γ‐secretase cleaves APP, it can produce amyloid peptides with different chain lengths, including Aβ37, 38, 39, 40, 42, and 43 (Pravin and Jozwiak 2022; Ratan et al. 2023). Aβ42 and Aβ40 are the two major Aβ species found in the brain. Due to the hydrophobicity of its two terminal residues, soluble Aβ42 has a greater tendency to aggregate, even though soluble Aβ40 is far more prevalent. Additionally, the primary constituent of amyloid plaques, Aβ42, has been demonstrated to be neurotoxic (Blennow and Zetterberg 2018). Aβ42 is therefore believed to be a crucial factor in the initiation of plaque formation and AD pathogenesis (Schützmann et al. 2021).

The formation of Aβ aggregates from Aβ monomers, including a variety of unstable oligomeric forms, is a pathophysiological aspect of AD. Short, elastic, irregular protofibrils are then formed by additional aggregation of oligomeric Aβ (oAβ). Over time, they develop into insoluble fibrillar assemblies composed of β‐strand repetitions oriented perpendicular to the fiber axis. The ability of Aβ peptides to self‐aggregate results in neurotoxicity (Aleksis et al. 2017; Fišar 2022). The toxicity of Aβ monomers is attributed to their propensity to oligomerize, forming protofibrils and fibrils. These microscopic pieces have the potential to induce excitotoxicity, inflammation, mitochondrial dysfunction, oxidative stress, and impaired membrane permeability. Additionally, Aβ disrupts membrane proteins, receptors, and channels, which hinders cell signaling (Savelieff et al. 2019; Sehar et al. 2022).

### 5.2 Tau Hyperphosphorylation

Tau, a microtubular protein, is mostly found in the axons and, to a lesser extent, glial cells and somatodendritic compartments. It is responsible for preserving the stability of the microtubule assembly, which is essential for axonal growth and neuronal development. Nevertheless, post‐translational modifications, such as phosphorylation at multiple sites, regulate its biological function (Burtscher et al. 2021). The MAPT gene on chromosome 17 encodes human tau and consists of 16 exons (Kent et al. 2020). In the human brain, up to six distinct tau isoform variants are generated by alternative splicing at exons 2, 3, and 10 (Wegmann et al. 2021). Protein kinases and protein phosphatases 1 and 2A (PP1 and PP2A) enzymes, associated with microtubules, control the addition and removal of phosphate groups from tau proteins. According to Rawat et al. (2022) and Congdon and Sigurdsson (2018), these enzymes control the equilibrium of tau protein phosphorylation and dephosphorylation in normal neural conditions. However, this equilibrium becomes unstable under pathological conditions, as kinases are upregulated and phosphatases are downregulated (Rahman et al. 2024). Tau proteins are thereby hyperphosphorylated, resulting in the formation of clusters and insoluble filaments known as neurofibrillary tangles (NFTs) (Fatemeh et al. 2023; Michalicova et al. 2020). NFT, which is the second most common histopathological characteristic of AD, is formed by the aggregation of tau protein incorporated within neurons (Kanaan et al. 2015; Savelieff et al. 2019). An aberrant tau phosphorylation increases the protein's propensity to assemble and decreases its affinity for microtubules. Thus, abnormalities in microtubule formation, axonal trafficking, and dendritic structure, as well as synaptic loss, neuronal death, and dementia, result from tau hyperphosphorylation, which also causes a loss of its fundamental function (Limorenko and Lashuel 2022). Prefibrillar aggregates, like Aβ, are the cause of tau‐mediated neurotoxicity. After aggregating to form oligomers, phosphorylated tau eventually develops into paired helical and straight filaments (Rahman et al. 2024).

Protein kinase A (PKA), calcium and calmodulin‐dependent protein kinase‐II (CaMKII), cyclin‐dependent protein kinase‐5 (cdk5), glycogen synthase kinase‐3 (GSK‐3), and other kinases phosphorylate tau in around 30 serine/threonine residues in AD (Dujardin et al. 2020; Laurent et al. 2018). Serine–threonine kinase GSK3 isoform β (GSK‐3β) has broad specificity and is an essential kinase that contributes to the tau hyperphosphorylation in AD. Therefore, like BACE‐1, it has been regarded as a promising therapeutic target for inhibiting or slowing disease progression (Souder and Anderson 2019; Yang et al. 2017).

Additionally, it has been noted that aging‐related increases in oxidative stress, compromised endoplasmic reticulum protein‐folding function, and abnormalities in proteasome‐mediated and autophagy‐mediated removal of damaged proteins might hasten the buildup of tau and amyloid proteins in AD (Lim et al. 2023; Liu et al. 2022). NFTs aggregate and develop as a result of these variables, ultimately leading to cell death and neural dysfunction.

### 5.3 Mitochondrial Dysfunction

Numerous investigations have suggested reduced mitochondrial activities in AD, particularly Ca^2+^ dysregulation, oxidative stress, mitochondrial DNA damage, altered mitochondrial dynamics, mitochondrial membrane permeabilization, and mitochondrial bioenergetics failure (Wong et al. 2020). The “powerhouse of cells,” mitochondria, is where energy is produced primarily through the electron transport chain (ETC) and the tricarboxylic acid cycle (TCA), which employ fatty acids, proteins, and glucose as energy sources. Acetyl‐CoA, which enters the TCA cycle to produce two kinds of electron donors, including nicotinamide adenine dinucleotide (NADH) and flavin adenine dinucleotide (FADH2), is produced when the metabolites undergo catabolism. Through the ETC, the site of oxidative phosphorylation (OXPHOS), NADH and FADH2 release energy and drive the intermembrane proton‐pumping mechanism, which generates an electrochemical gradient. The ATP synthase eventually uses the energy from the proton gradient to produce ATP. Because numerous redox reactions occur during this process, a significant quantity of ROS is produced as by‐products. As reported, excess ROS generation impairs TCA and ETC in AD, which has been linked to oxidative stress, reduced antioxidants, impaired translocase activity, and dysregulated mitochondrial enzymes (Martínez et al. 2020). In AD, functional deficiencies in the ETC result in decreased ATP production and mitochondrial membrane potential (MMP), which affect mitochondria's capacity to meet the cell's active needs. Multiple studies across cell types have demonstrated that Complex IV is less active in AD, and other electron transport chain (ETC) proteins also exhibit compromised function (Afsar et al. 2023; Lopez Sanchez et al. 2017).

It remains unknown whether mitochondria have innate calcium homeostasis deficiencies distinct from tau‐ and amyloid‐mediated dysfunction, as the pathological processes of AD impair mitochondria's capacity to store and buffer calcium (Sarasija et al. 2018). Higher levels of cellular and mitochondrial oxidative stress are caused by AD mitochondria's increased propensity to generate ROS, which further impairs ETC function as the disease worsens (Bell et al. 2021). At the onset of AD, both the brain and peripheral cells exhibit elevated mitophagy, which progressively declines as the disease progresses (Cai and Jeong 2020; Kerr et al. 2017). This discovery led to the development of the mitochondrial cascade theory, which postulates that increased Aβ synthesis and plaque deposition are significantly influenced by altered mitochondrial activity (Jörg et al. 2021). A prospective association between Aβ40 and mitochondrial dysfunction was suggested by Casoli et al. who reported a 73% increase in Aβ40 levels in the test group, along with enlarged mitochondria (Casoli et al. 2007). In another study, Jadiya et al. demonstrated that NCLX‐cKO × 3xTg‐AD mice exhibited signs of AD pathogenesis, including calcium dysregulation, a 60% increase in amyloid deposits, and a decline in spatial memory (Jadiya et al. 2019). As a result, changes in mitochondrial dynamics in AD may underlie changes in mitochondrial function. Still, cellular exposure to tau and amyloid aggregates in AD also impacts (Singh et al. 2024).

### 5.4 Oxidative Stress

The inefficiency of antioxidant defenses results in an imbalance between oxidant generation and elimination, known as oxidative stress. As a result, it has become more prevalent for high concentrations of reactive species produced by nitrogen and oxygen to accumulate. The primary effect of this imbalance is the oxidative modification of nucleic acids, proteins, and lipids (lipid peroxidation) (Forman and Zhang 2021; Ito et al. 2019). One such study, for instance, showed a link among protein oxidation, nucleic acid oxidation, and lipid peroxidation. Malondialdehyde (MDA), a marker of lipid peroxidation, showed a marked decrease in expression, supporting the idea that oxidative stress induced by lipid peroxidation is a pathogenic process in Alzheimer's disease (Wharton et al. 2023). Furthermore, many studies have demonstrated that oxidative stress promotes the development of senile plaques by reducing α‐secretase activity and increasing β‐ and γ‐secretase activities (Ganguly et al. 2017; Hajam et al. 2022). Furthermore, it has been observed that Aβ can directly activate NADPH oxidase, generate a concentration‐dependent accumulation of ROS, and boost its production. This means that oligomeric buildup exacerbates oxidative stress (Bai et al. 2022; Ma et al. 2017).

Activated glial cells are another histopathological indicator of AD, with neuroinflammation present in brain regions affected by the condition. This is a natural physiologic reaction of the central nervous system to the buildup of Aβ and hyperphosphorylated Tau (Ali et al. 2024; Ju and Tam 2022). Additionally, oxidant species can promote the transcription of proinflammatory genes in astrocytes and microglia. The inflammatory response in AD brains is triggered by the production of ROS and proinflammatory cytokines, such as interleukin‐6 (IL‐6), IL‐1β, and tumor necrosis factor‐α (TNF‐α), by activated microglial cells. Additionally, Aβ synthesis and accumulation are favored by IL‐1, IL‐6, and TNF‐α (Kwon and Koh 2020). Oxidative stress‐activated reactive astrocytes and microglia can cause synaptic loss, increase Tau pathology by upregulating kinases that hyperphosphorylate Tau, contribute to the production and accumulation of Aβ, and produce proinflammatory cytokines that cause neuronal death via apoptosis (Perez‐Nievas and Serrano‐Pozo 2018; Walker et al. 2019).

### 5.5 Ferroptosis

The accumulation of iron, the most common redox‐active metal in the human body, in the brain is a defining feature of neurological disorders. In AD, iron buildup is in control of: (i) producing reactive oxygen species (ROS), which causes multiple cellular damage; (ii) promoting Aβ aggregation and oligomerization, which contributes to Aβ toxicity; and (iii) taking part in the hyperphosphorylation and subsequent aggregation of Tau protein (Nakamura et al. 2019). An iron‐dependent form of cell death induced by oxidative stress is called ferroptosis, which occurs when the antioxidant GSH‐dependent system becomes inactive, allowing harmful lipid‐ROS to accumulate (Cao and Dixon 2016; Han et al. 2020).

One of the main symptoms of AD is brain atrophy. Patients with AD often have smaller medial‐temporal brain volumes, particularly in the hippocampus, which is closely linked to their decreased overall cognitive function (Pini et al. 2016; Uysal and Ozturk 2020). Interestingly, iron accumulation sites were strongly correlated with brain regions that exhibited neuronal degeneration and atrophy (Horvath et al. 2012). These results strongly imply that excessive iron buildup is associated with AD interferonopathies.

Ferroptosis was similarly characterized by the accumulation of large lipid peroxides to deadly levels, and the pathophysiology of AD likewise showed enhanced lipid peroxide indicators. Clinically, metabolites of lipid peroxidation co‐localized with amyloid plaques and were strongly associated with the advancement of AD (Yin 2023). Furthermore, the hippocampal regions of AD patients showed markedly lower levels of total fatty acids derived from membrane phospholipids [phosphatidylethanolamine (PE) and phosphatidylinositol (PI)] (Stockwell et al. 2017). Additionally, AD‐related decreases in GSH levels have been noted in both animal models (Zhang et al. 2012) and human brains (Chiang et al. 2017). Notably, brain amyloidosis and AD pathology were closely correlated with GSH levels. Consequently, ferroptosis was likely caused or enhanced by the altered iron distribution in AD, which may have contributed to the disease's neuronal death and degeneration (Li et al. 2020; Yu et al. 2021).

## 6 Biosurfactant as a Novel Therapy for the Treatment of Alzheimer's Disease

Alzheimer's disease is a progressive neurodegenerative disease characterized by memory loss, synaptic dysfunction, and cognitive decline. Various factors, including aggregation of amyloid‐beta, hyperphosphorylation of tau, oxidative stress, and neuroinflammation, contribute to the pathogenesis of AD. Current treatments for AD provide only symptomatic relief, necessitating novel therapeutic approaches (Zhang et al. 2024). In this context, biosurfactants, amphiphilic compounds produced by microorganisms, have emerged as potential candidates due to their diverse biological activities in mitigating key pathological features of AD (Rout et al. 2024).

The development of AD is significantly influenced by the aggregation of amyloid beta peptides into oligomers and fibrillar plaques that disrupt neuronal function (Azargoonjahromi 2024). Biosurfactants can interact with cellular membranes and protein aggregates because they possess both hydrophilic and hydrophobic domains. This amphiphilic nature may allow biosurfactants to interfere with the formation of amyloid‐beta fibrils by disrupting peptide aggregation and enhancing the solubilization of amyloid‐beta aggregates, resulting in their degradation and clearance (Purohit et al. 2024). These interactions imply that biosurfactants may reduce amyloid‐induced neurotoxicity.

Oxidative stress and neuroinflammation significantly contribute to neuronal damage in AD (Firdous et al. 2024). Biosurfactants have been reported to exhibit potent anti‐inflammatory properties through the regulation of pro‐inflammatory cytokines such as interleukin‐6 (IL‐6) and tumor necrosis factor‐α, and the inhibition of nuclear factor‐κB signaling. This alteration can limit neuroinflammatory damage in neural tissues and reduce microglial activation (Lawal et al. 2022). In addition, a few biosurfactants, including glycolipids, have been reported to possess antioxidative properties that may help neutralize reactive oxygen species and prevent oxidative neuronal damage (Kumari et al. 2023).

**FIGURE 5:**
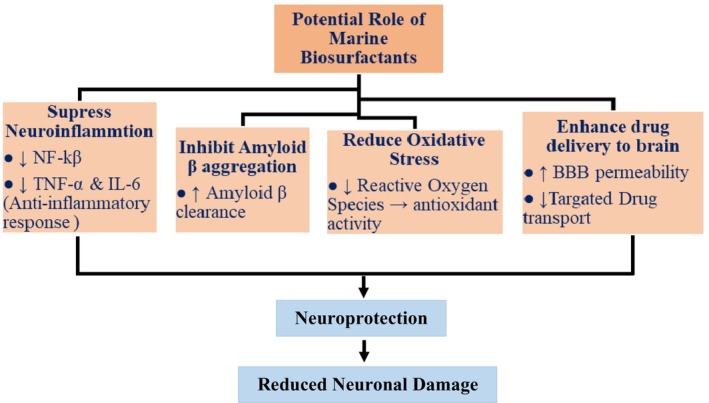
Schematic representation of potential neuroprotective mechanisms of biosurfactants in Alzheimer's disease, including inhibiting amyloid‐β aggregation and promoting its clearance, reducing oxidative stress, and enhancing drug delivery across the blood–brain barrier.

The restricted ability of therapeutic agents to cross the blood–brain barrier is another major challenge in AD therapy (Iqbal et al. 2024). Due to their emulsifying and membrane‐permeabilizing properties, biosurfactants can be proposed as potential drug delivery agents that may enhance drug bioavailability and facilitate targeted delivery of compounds used for AD treatment (Dini et al. 2024; Hernandez and Shukla 2022). Additionally, the neuroprotective potential of biosurfactants may vary by class. The membrane‐interacting and anti‐inflammatory properties of lipopeptide biosurfactants, mostly derived from *Bacillus* and marine actinomycetes, are notable. Glycolipid biosurfactants, such as sophorolipids, are reported to exhibit antioxidant properties relevant to neuroprotection. Although there aren't many comparative studies on their effectiveness in AD, lipopeptides, in particular, show promise in modulating inflammation and interacting with protein aggregates. A schematic representation of the possible mechanisms by which biosurfactants may have neuroprotective effects in AD (Figure 5). Additionally, Table 3 provides a comparative overview of major biosurfactant classes and their potential relevance 0to AD treatment (Park et al. 2013a, 2013b).

**TABLE 3:** Comparative potential of different classes of biosurfactants in AD.

Biosurfactant class	Major microbial source	Key biological activity	Potential relevance to AD	References
Lipopeptides	*Bacillus* sp.	Anti‐inflammatory	May inhibit amyloid aggregation and reduce neuroinflammation	Dini et al. 2024
Glycolipids (Rhamnolipids)	*Pseudomonas* sp.	Antioxidant	May reduce oxidative stress	Dini et al. 2024
Glycolipids (Sophorolipids)	Yeasts	Anti‐inflammatory. ROS reduction	Potential neuroprotective effects	Giri et al. 2020

Thus, biosurfactants offer a novel therapeutic approach for AD by preventing the aggregation of amyloid‐beta, reducing inflammation and oxidative stress, and enhancing drug delivery. However, more investigation is required to refine the formulations and assess their safety, effectiveness, and therapeutic suitability in the treatment of AD.

## 7 Conclusion

Biosurfactants present a promising strategy for treating AD, complex neurodegenerative diseases marked by amyloid‐beta aggregation, tau protein hyperphosphorylation, oxidative stress, and neuroinflammation. Current treatment options primarily provide symptomatic relief without stopping disease progression, highlighting the need for novel alternatives. Research indicates that biosurfactants derived from microorganisms exhibit unique biological properties, including anti‐inflammatory and antioxidant effects that may enhance neuroprotection through mechanisms such as inhibition of amyloid‐beta aggregation, reduction of oxidative stress, suppression of neuroinflammation, and modulation of membrane interactions and signaling pathways. Their amphiphilic properties can enhance drug delivery by improving solubility and bioavailability, potentially facilitating targeted delivery across the blood–brain barrier. Given that existing knowledge is primarily rooted in their general biological functions and potential uses in biomedical and drug delivery systems, current research lacks direct experimental studies on the therapeutic effects of biosurfactants in AD. Significant research gaps persist, particularly regarding the mechanisms underlying interactions between biosurfactants and amyloid‐beta peptides, tau pathology, and neuronal signaling pathways. However, further research is needed to assess their mechanisms of action, bioavailability, safety profiles, and the ability of biosurfactants to penetrate the blood–brain barrier. Integration of biosurfactants with advancements in nanomedicine may also improve the efficacy of biosurfactant‐based therapies. Ultimately, biosurfactants may serve as sustainable and biocompatible agents for AD management, yet comprehensive experimental, preclinical, and clinical research is essential to assess their efficacy in AD treatment.

## Author Contributions

**Sumon Giri:** data curation, writing – review and editing. **Shakti Ketan Prusty:** supervision, writing – review and editing. **Arijit Nandi:** writing – review and editing, data curation. **Mostafa Gouda:** conceptualization, writing – original draft, writing – review and editing, supervision, data curation. **Zainab Irfan:** writing – review and editing, writing – original draft, data curation, conceptualization.

## Funding

The authors have nothing to report.

## Ethics Statement

The authors have nothing to report.

## Conflicts of Interest

The authors declare no conflicts of interest.

## Data Availability

No data was used for the research described in the article.
